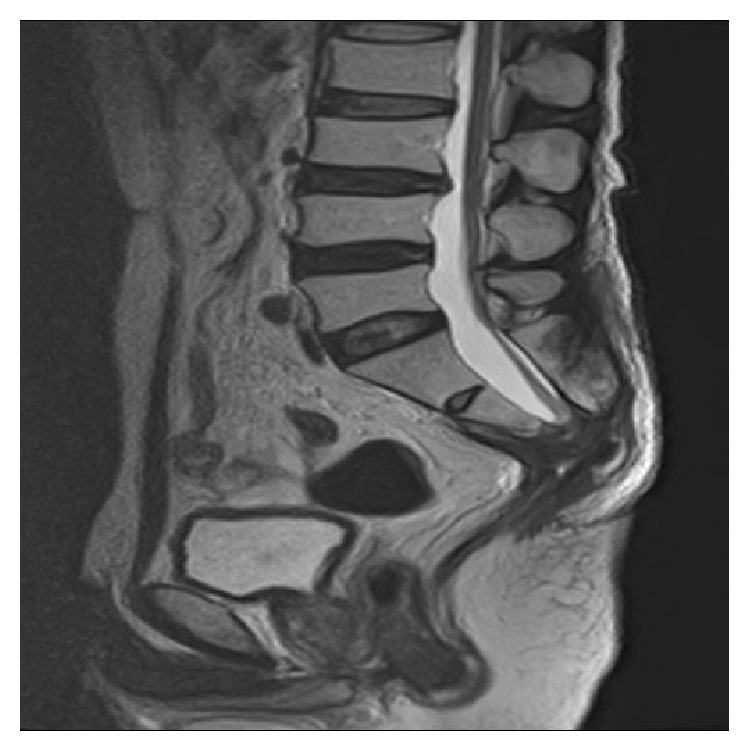# An Unusual Presentation of Adult Tethered Cord Syndrome Associated with Severe Chest and Upper Back Pain

**DOI:** 10.1155/2015/926185

**Published:** 2015-09-09

**Authors:** Shotaro Kanda, Toru Akiyama, Hirotaka Chikuda, Takehiko Yamaguchi, Kazuo Saita

**Affiliations:** ^1^Department of Orthopaedic Surgery, Saitama Medical Center, Jichi Medical University, 1-847 Amanuma, Omiya-ku, Saitama 330-8503, Japan; ^2^Department of Orthopaedic Surgery, Faculty of Medicine, The University of Tokyo, 7-3-1 Hongo, Bunkyo-ku, Tokyo 113-8655, Japan; ^3^Department of Pathology, Dokkyo Medical University Koshigaya Hospital, No. 1-50, 2-Chome, Minami-Koshigaya, Koshigaya-shi, Saitama 343-8555, Japan

## Abstract

Adult tethered cord syndrome (ATCS) is a rare entity that usually presents with multiple neurological symptoms, including lower extremity pain, backache, lower extremity muscle weakness, and bowel/bladder disturbances. Prompt surgical treatment is often necessary to avoid permanent sequelae. We report a 63-year-old man with sudden-onset severe right chest and upper back pain, followed by urinary retention. His initial workup included computed tomography of the abdomen and pelvis, which showed a presacral mass. His symptom-driven neurological workup focused on the cervical and thoracic spine, the results of which were normal. Pelvic radiographs and magnetic resonance imaging of the lumbosacral spine showed spina bifida occulta, meningocele, and presacral masses consistent with a teratomatous tumor. His symptoms, except for urinary retention, improved dramatically with surgical treatment. The excised specimen contained a teratomatous lesion plus an organized hematoma. Hematoma formation was suspected as the trigger of his sudden-onset right chest and upper back pain.

## 1. Introduction

Tethered cord syndrome (TCS) usually presents at birth or during childhood with symptoms including back pain radiating to both buttocks and legs, sensory deficit of the lower extremities, and loss of bladder/bowel control [1–3]. Adult tethered cord syndrome (ATCS) is a rare entity, and the diagnosis is still challenging [3, 4]. Pain in the lower back or legs is the predominant symptom [2, 5, 6]. Sensory disturbances are often patchy in distribution rather than adhering to a particular dermatome [3]. Pain in the upper limbs and upper trunk is rare. Chest pain in a patient with ATCS has never been reported in the English literature. Because the lesion location does not match the presenting symptoms, ATCS with predominantly upper body neurologic abnormalities may be misdiagnosed. We report a 63-year-old man with sudden-onset severe right chest and upper back pain, followed by urinary retention, who was diagnosed with ATCS.

## 2. Case Presentation

A 63-year-old man was brought to our hospital by ambulance with urinary retention and severe right chest and right upper back pain. Thirteen days prior to admission, he suddenly developed right chest and upper back pain while washing his car. He sought help at a nearby hospital. No diagnosis was made, and he was discharged with pain medication. Ten days later, urinary retention occurred, and he returned to the hospital. Bladder catheterization drained 800 mL of urine. Computed tomography (CT) of the abdomen and pelvis showed a presacral mass, and he was emergently referred to our hospital.

He had no history of neurological problems in childhood. His family history was noncontributory. He could not walk because of intermittent severe right chest and right upper back pain. He had mild lower leg pain. On physical examination, he had no cutaneous abnormalities at the lumbosacral level. Paresthesia of the right side of the body from the shoulders down was noted. Pain and temperature sensation on the left side of the body below T2-3 were reduced. There were no cranial nerve abnormalities. Muscle strength assessed by manual muscle testing was 3/5 in the right arm and 4/5 in the right leg. Muscle strength in the left upper and lower extremities was normal. Both patellar tendon reflexes were increased; the other deep tendon reflexes were normal.

**Figure 1:**
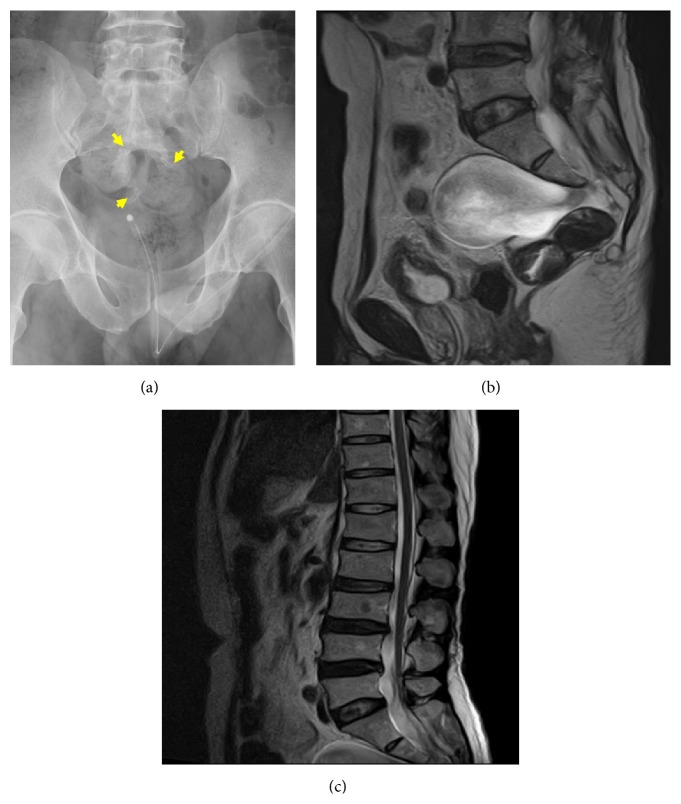


The bladder was catheterized, draining 800 mL of urine. There was reduced anal sphincter tone with fecal incontinence. CT and magnetic resonance imaging (MRI) of the cervicothoracic spine showed no obvious abnormalities. A radiograph of the pelvis showed a hook-shaped defect in the left sacral body (Figure 1(a)).

MRI showed a bony defect of the anterior sacrum, with a large mass arising from the dura, which was associated with two smaller masses (Figure 1(b)). T2-weighted images showed a homogeneous high signal in the large mass, which was characteristic of a meningocele. A low-lying conus medullaris, below the level of L4-L5, was also noted (Figure 1(c)).

Sixty milligrams of codeine phosphate provided pain relief, enabling the patient to walk with a T-cane 6 days after admission. The patient's pain improved from a 10/10 on the numeric rating scale at admission to a 4/10 with the administration of codeine 2 days later. He was able to defecate with the help of a laxative, but urinary retention persisted.

CT-guided biopsy was performed. The pathological diagnosis of both the small presacral masses was consistent with teratoma. We conjectured that his symptoms were triggered by TCS associated with spina bifida occulta, meningocele, and presacral tumors.

His clinical condition was similar to the observations in Currarino syndrome, although they did not fulfill the triads of Currarino syndrome (i.e., sacral agenesis, a presacral mass, and anorectal malformation). His family did not present with similar abnormalities. Currarino syndrome-specific mutations in homeobox gene,* HLXB9*, have been reported [7]. Genetic mutation analysis was not performed in our patient, because his clinical condition was not Currarino syndrome.

We performed presacral tumor resection at the level of S2-3 and untethered the cord using a posterior sacral approach. After laminectomy of S2 and S3, an incision was made in the dura mater. We confirmed the filum terminale and S2 and S3 roots on both the sides. The roots above S3 in the surgical field were not abnormally strained.

**Figure 2:**
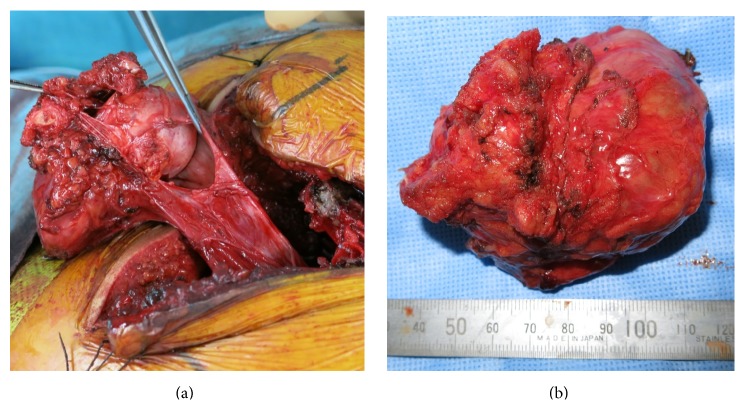


The S3 nerve roots could not be divided from the presacral masses; they were not connected to the meningocele. After confirming that they were not involved with contraction of the anal sphincter with electrical nerve stimulation (Figures 2(a) and 2(b)), the masses with both S3 roots and the meningocele were resected en bloc. To untether the conus, we resected the filum terminale. After performing the untethering procedure with resection of the filum terminale, the orifice was closed by oversewing with interrupted nylon 4-0 sutures.

**Figure 3:**
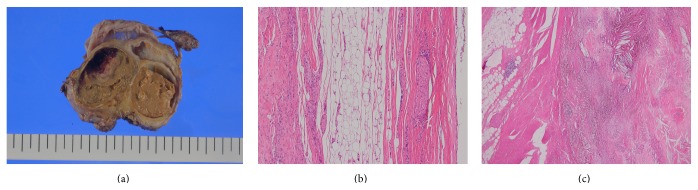


The excised specimen consisted of a fibrous soft tissue mass measuring approximately 7 × 5 cm at its greatest dimension. The cut surface had two cystic lesions with thick fibrous walls, measuring up to approximately 4 × 3 cm in size. The cysts were filled with yellowish-brown, turbid material, and the larger cyst contained a hematoma measuring 2.5 × 2 cm (Figure 3(a)). Microscopically, the cysts were lined by squamous or columnar epithelium that was often attenuated. The walls were composed of fibroadipose tissue intermingled with numerous peripheral nerve fibers (Figure 3(b)). These findings mimic teratoma but are consistent with the anomalous tissue associated with spina bifida. In addition, a hematoma was recognized in the cyst (Figure 3(c)). It was associated with organizing granulation tissue with cholesterol clefts and hemosiderin pigment deposits at the periphery. The findings were suggestive of organized hematoma from approximately 1 month ago.

The patient was able to walk unaided at discharge 6 weeks postoperatively. He did not require any analgesic drugs. A follow-up MRI demonstrated no residual tumor, but the conus remained at L4-L5 (Figure 4). Eighteen months postoperatively, his gait normalized, but he continues to require self-catheterization and enemas to facilitate bowel movement. Paresthesia in the right fourth and fifth fingers and the right lower leg persists.

## 3. Discussion

Diagnosis and treatment of ATCS are a challenging task because of its rarity, and its presentation mimics a wide spectrum of pathologies [3, 4, 8]. The clinical features of ATCS are widely recognized as nonspecific, including diffuse and nondermatomal back and leg pain [2, 3, 9–11]. Other common symptoms include bladder dysfunction, skin abnormalities, scoliosis, and decreased motor and sensory function [2, 10, 11]. Demonstration of the position of the conus below L2, a thickened filum terminale, diastematomyelia, or a lipoma with extradural extension is essential for the diagnosis of a tethered cord [8].

In our case, the predominant symptoms contributing to the diagnosis were right upper back and chest pain and paresthesia of the entire right side of the body from the shoulders down. To our knowledge, only two cases of ACTS presenting with upper extremity pain and numbness have been reported [11, 12]. One case presenting with upper extremity pain had cervical cord lesions [11].

Prompt surgery is recommended for patients with symptomatic ATCS without complex malformations such as teratomas [2, 5, 8]. For ATCS patients with complex malformations, prompt surgery is advised for avoiding symptom progression. Surgery was reported to result in a good overall outcome in 61–85% of patients [5, 8]. Improvement in pain and lower extremity weakness has been reported in 65–83% and 46%–78% of patients, respectively [5, 7, 10, 11]. Bladder disturbances were less responsive to surgical treatment, showing improvement in 46%–50% patients [3, 5, 6, 10]. Morbidity is reported to range from 12% to 19% [2, 3, 5, 8]. Cerebral spinal fluid (CSF) leakage was the most common postoperative complication [2, 3, 5, 6, 8]. Infections following CSF leakage and/or pseudomeningocele formation varied from 0% to 33% [2, 5, 6, 8, 11]. It should be noted that 3.5% of patients will experience significant deterioration, and 3.3–16% will require repeat detethering [5, 6]. The long-term prognosis is clearly related to the presence or absence of symptomatic retethering [8]. The clinical recurrence rate and postoperative morbidity are much higher in ATCS with complex malformations than in uncomplicated ATCS [8].

The postoperative outcome of ATCS may be worse in patients with upper extremity symptoms than in those with low back pain, lower extremity symptoms, or both [11, 12]. Some improvement was shown in both reported cases, although one patient had recurrence of tethering and progression of symptoms [11, 12]. MRI is not a reliable postoperative diagnostic modality because there is no significant ascent of the conus after operation in most patients despite neurological improvement and a good outcome [3]. In our patient, MRI did not show a significant ascent of the conus postoperatively, but the postoperative outcome was good.

Our patient demonstrated improvement in the right upper chest and back pain and right-sided body paresthesia postoperatively, but there was no improvement in bowel incontinence or urinary retention. Upper trunk pain was the presenting symptom in the two reported cases with upper extremity symptoms [11, 12]. This unusual presentation of ATCS should be kept in mind when treating a patient with diverse nonspecific symptoms, especially when accompanied by fecal incontinence and urinary retention.

The onset of right chest and upper back pain was about 1 month prior to his presentation at our hospital, suggesting that the hematoma formation coincided with the onset of symptoms. Hematoma formation or hemorrhage was suspected as a trigger of his sudden onset of right chest and upper back pain, although hemorrhage has not been reported as a trigger of TCS.

## Figures and Tables

**Figure 4:**